# mTOR Pathway and mTOR Inhibitors in Head and Neck Cancer

**DOI:** 10.5402/2012/953089

**Published:** 2012-10-18

**Authors:** Wei Gao, John Zeng Hong Li, Jimmy Yu Wai Chan, Wai Kuen Ho, Thian-Sze Wong

**Affiliations:** Department of Surgery, The University of Hong Kong and Queen Mary Hospital, 102 Pokfulam Road, Hong Kong

## Abstract

Head and neck cancer is the sixth most common type of Cancer worldwide. Since conventional treatment regimens are nonselective and are associated with systemic toxicities, intense investigations focus on molecular targeted therapy with high selectivity and low adverse effects. mTOR signaling pathway has been found to be activated in head and neck cancer, making it attractive for targeted therapy. In addition, expression levels of mTOR and downstream targets eIF4E, 4EBP1, S6K1, and S6 are potential diagnostic and prognostic biomarkers for head and neck cancer. mTOR inhibitors, such as rapamycin and its derivatives temsirolimus and everolimus, exhibit inhibitory effects on head and neck cancer in both *in vitro* cell line model and *in vivo* xenograft model. A large number of clinical trials have been initiated to evaluate the therapeutic effects of mTOR inhibitors on patients with head and neck cancer. mTOR inhibitor has potential as a single therapeutic agent or in combination with radiation, chemotherapeutic agents, or other targeted therapeutic agents to obtain synergistic repression on head and neck cancer.

## 1. Introduction

Head and neck cancer is the sixth most common type of cancer worldwide, with about 650,000 new cases in the world every year [1]. Tobacco and alcohol consumption is a main risk factor for head and neck cancer [1]. In addition, accumulating evidence has shown that human papillomavirus and Epstein-Barr virus are associated with carcinogenesis in oropharyngeal cancers and nasopharyngeal cancers, respectively [2, 3]. The treatment methods for head and neck cancer include surgery, radiotherapy, and chemotherapy [4]. Patients with early stages of disease are treated by surgery and radiotherapy, while patients with advanced stages of disease are administrated by surgery and chemoradiotherapy [4]. Platinum-based agents (cisplatin/carboplatin), taxane agents (docetaxel/paclitaxel), and 5-fluorouracil are the most common chemotherapeutic agents for head and neck cancer [4–7] (Table 1). 

Despite advances in treatment methods for head and neck cancer, the survival rate has not been largely improved [4]. The major reason is that conventional treatment regimens are nonselective and are related with systemic toxicities [8, 9]. Therefore, intense investigations focus on alternative treatment strategies with less systemic toxicities for head and neck cancer. Since molecular targeted therapy has high selectivity and low adverse effects, it exhibits promise as an alternative treatment strategy for head and neck cancer. Multiple molecular signaling pathways have been found to be dysregulated in head and neck cancer, making it attractive for targeted therapy. 

**Figure 1:**
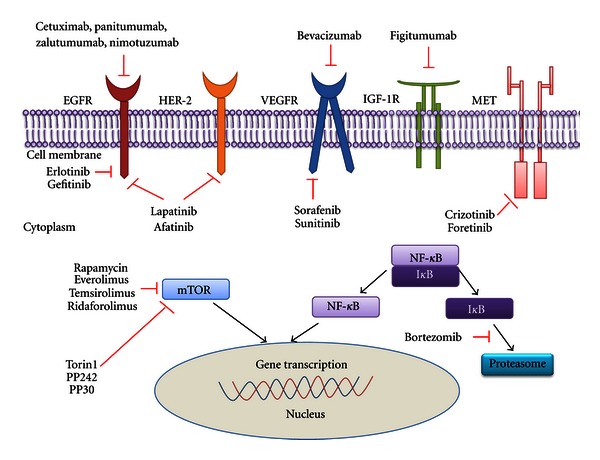
Targeted therapies for head and neck cancer. EGFR: epidermal growth factor receptor; HER-2: human epidermal growth factor receptor 2; VEGFR: vascular endothelial growth factor receptor; IGF-1R: insulin growth factor-1 receptor; mTOR: mammalian target of rapamycin; NF-*κ*B: nuclear factor-kappa B.

 Targeted therapy focuses on oncogenic signaling pathways involved in carcinogenesis of head and neck cancer, such as epidermal growth factor receptor (EGFR), human epidermal growth factor receptor 2 (HER-2), vascular endothelial growth factor receptor (VEGFR), insulin growth factor-1 receptor (IGF-1R), MET receptor, transcriptional factor nuclear factor-kappa B (NF-*κ*B), and phosphatidylinositol-3-kinase (PI3K)/AKT/mammalian target of rapamycin (mTOR) pathway [10, 11]. Clinical and preclinical investigations have developed some promising targeted agents for head and neck cancer including EGFR inhibitors (cetuximab, panitumumab, zalutumumab, nimotuzumab), EGFR tyrosine kinase inhibitors (gefitinib, erlotinib), dual EGFR/HER-2 kinase inhibitors (lapatinib, afatinib), VEGFR inhibitor (bevacizumab), VEGFR tyrosine kinase inhibitors (sorafenib and sunitinib), IGF-1R inhibitor (figitumumab), MET tyrosine kinase inhibitors (crizotinib, foretinib), mTOR inhibitors (rapamycin, everolimus, temsirolimus, ridaforolimus, Torin1, PP242, and PP30), and proteasome inhibitor (bortezomib) (Figure 1) [10, 11]. 

 mTOR signaling pathway has been found to be activated and expression levels of mTOR and downstream proteins are potential diagnostic and prognostic biomarkers for head and neck cancer. Furthermore, mTOR inhibitors exhibit inhibitory effects on head and neck cancer. Therefore, this paper emphasizes on dysregulated mTOR signaling pathway and the role of mTOR inhibitors in head and neck cancer.

## 2. mTOR Signaling Pathway in Head and Neck Cancer

**Figure 2:**
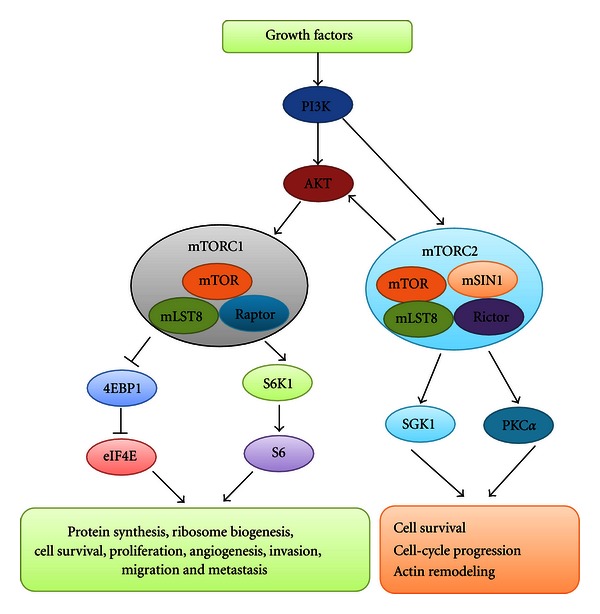
mTOR signaling pathway. PI3K: phosphatidylinositol-3-kinase; mTOR: mammalian target of rapamycin; mTORC1: mTOR complex 1; mTORC2: mTOR complex 2; Raptor: regulatory-associated protein of mTOR; Rictor: rapamycin-insensitive companion of mTOR; mSIN1: mammalian stress-activated protein kinase interacting protein; eIF4E: eukaryotic initiation factor 4E; 4EBP1: eIF4E -binding protein 1; S6K1: S6 kinase 1; PKC*α*: protein kinase C*α*; SGK1: glucocorticoid-induced protein kinase 1.

mTOR is an important downstream signal of PI3K/AKT/mTOR signaling pathway and it is activated in head and neck cancer [12]. The structure of mTOR contains N-terminal tandem HEAT repeats, FAT domain, FATC domain, FRB domain, and C-terminal kinase domain [12]. There are two distinct mTOR complexes designated as mTOR complex 1 (mTORC1) and mTOR complex 2 (mTORC2) (Figure 2) [13, 14]. mTORC1 is composed of mTOR, regulatory-associated protein of mTOR (Raptor) and mLST8 [15]. mTORC2 is comprised of mTOR, rapamycin-insensitive companion of mTOR (Rictor), mLST8 and mammalian stress-activated protein kinase interacting protein (mSIN1) [14]. mTORC1 is a nutrition- and rapamycin-sensitive complex, while mTORC2 is insensitive to rapamycin [16, 17].

 mTORC1 is regulated by multiple signals, such as growth factors, nutrients, energy status, and oxygen and cellular stress [13, 14]. mTORC1 promotes protein synthesis, proliferation, cell survival, ribosome biogenesis, angiogenesis, migration, invasion, and metastasis by phosphorylation of ribosomal protein S6 kinase 1 (S6K1) and eukaryotic initiation factor 4E (eIF4E)-binding protein 1 (4EBP1) (Figure 2) [16, 18–20]. The phosphorylation of 4EBP1 results in the release of eIF4E. Subsequently, the free eIF4E enhances the translation of c-myc, cyclin D1, VEGF, and matrix metalloproteinase-9 (MMP-9), thus promoting cell survival, angiogenesis, invasion and metastasis [21–23]. The activation of S6K1 promotes ribosome biogenesis *via* upregulation of ribosomal protein S6 [24]. mTORC2 functions in actin remodeling, cell-cycle progression, and cell survival through the regulation of protein kinase C*α* (PKC*α*) and glucocorticoid-induced protein kinase 1 (SGK1) [17, 25].

 Some studies have demonstrated that expression levels of mTOR and downstream targets eIF4E, 4EBP1, S6K1, and S6 are potential diagnostic and prognostic biomarkers for head and neck cancer. In an experiment to evaluate mTOR protein expression in 25 patients with laryngeal carcinoma treated with postoperative radiotherapy, it was found that high expression of mTOR was a prognostic marker for high risk of recurrence after postoperative radiotherapy [26]. Elevated level of eIF4E in tumor-free surgical margins was correlated with local-regional recurrence in patients with head and neck cancer [27–29]. Increased level of phosphorylated active form of S6 was observed in cell lines and tumor tissues from patients with head and neck cancer [29–31]. The mRNA level of *4EBP1* was repressed, while the mRNA expression of *S6K1* was enhanced in tumors of patients with oral squamous cell carcinoma [29]. Expressions of phosphorylated S6K1 and phosphorylated 4EBP1 were regulated by LMP1 and were associated with overall survival of patients with nasopharyngeal carcinoma (NPC), indicating that they were potential prognostic biomarkers for NPC patients [32]. Clark et al. carried out an experiment to find the best molecular markers in the mTOR pathway for head and neck cancer [33]. It was found that phosphorylated mTOR exhibited better sensitivity and specificity than phosphorylated 4EBP1 in differentiating tumor from normal mucosa from patients with head and neck cancer [33]. 

## 3. mTOR Inhibitors in Head and Neck Cancer

mTOR signaling pathway was activated in head and neck cancer, making it attractive for targeted therapy. Two types of mTOR inhibitors designated as first-generation and second-generation inhibitors have been developed to interrupt mTOR [34]. First-generation mTOR inhibitors refer to rapamycin and its derivatives temsirolimus, everolimus, and ridaforolimus [14]. Second-generation mTOR inhibitors refer to ATP-competitive mTOR inhibitors including Torin1, PP242, and PP30 [34]. Rapamycin represses the kinase activity of mTOR1 by binding to the FKBP12-rapamycin (FRB) domain of mTORC1 [35]. Since rapamycin has poor water solubility, absorption, and low bioavailability [36], its derivatives are developed to improve bioavailability by a chemical modification at C-40-0 of rapamycin [37]. ATP-competitive mTOR inhibitors suppress the catalytic activities of both mTORC1 and mTORC2 by binding to the kinase domain [34]. 

### 3.1. Inhibitory Effects of Rapamycin on Head and Neck Cancer

Some studies have reported the inhibitory effects of rapamycin on head and neck cancer using both *in vitro* cell line model and *in vivo* xenograft model. Rapamycin suppressed growth of SCC-15 cells by inhibiting phosphorylation of mTOR *in vitro* [38]. Rapamycin prevented tumorigenesis of head and neck cancer in a 4-nitroquinoline-1 oxide carcinogenesis mice model and a k-ras and p53 two-hit carcinogenesis mice model [39, 40]. Amornphimoltham et al. found that rapamycin treatment significantly repressed tumor growth of nude mice bearing xenografts derived from human head and neck cancer cell lines HN21, CAL27, and UMSCC11B [30]. Moreover, it was found that rapamycin inhibited phosphorylation of S6, repressed DNA synthesis, and induced apoptosis in xenograft model [30]. 

### 3.2. Inhibitory Effects of Temsirolimus on Head and Neck Cancer

The therapeutic effects of temsirolimus on head and neck cancer have also been demonstrated in several studies in both *in vitro* cell line model and *in vivo* xenograft model. Temsirolimus treatment inhibited cell proliferation of head and neck cancer cell lines PCI-1 and PCI-13 *in vitro* [41]. An experiment carried out by Nathan et al. also reported that temsirolimus dose-dependently repressed the proliferation of head and neck cell lines FaDu and FaDu9000 by inhibiting phosphorylation of 4EBP1 *in vitro* [42]. Furthermore, temsirolimus-treated mice showed reduced tumor size, inhibited phosphorylation of S6, and decreased phosphorylation of 4EBP1 in comparison to control mice in xenograft model [42]. This study has also employed a minimal residual disease (MRD) model to further examine the efficacy of temsirolimus. The MRD model was performed to mimic patients with molecular positive margins. Mice treated by temsirolimus exhibited decreased proportion of mice with tumors, repressed tumor size, and retarded time to develop tumors in comparison with control mice in the MRD model [42]. Finally, it was found that temsirolimus treatment repressed the phosphorylation of 4EBP1 in peripheral blood mononuclear cells of mice from the MRD model, suggesting that the phosphorylation of 4EBP1 was a promising biomarker to monitor the response of tumors to temsirolimus [42]. Jimeno et al. showed that temsirolimus inhibited the growth of Detroit 562 cells to 13% in xenograft-bearing nude mice. Furthermore, temsirolimus-treated mice displayed a decrease in vessel intratumor growth, while control mice showed widespread vessels in the tumors [43]. These results suggested that temsirolimus inhibited both proliferation and angiogenesis in xenograft model.

### 3.3. Inhibitory Effects of Everolimus on Head and Neck Cancer

Several studies have also reported the anticancer effects of everolimus on head and neck cancer. Patel et al. found that everolimus and rapamycin inhibited phosphorylated S6 in the primary tumor site and metastatic lymph nodes of mice with UMSCC2-derived xenografts growing in the tongue [44]. In addition, mice treated by everolimus and rapamycin displayed areas of squamous differentiation and fibrosis, while control mice exhibited areas with active cell growth, indicating that everolimus and rapamycin treatment significantly repressed tumor growth [44]. Moreover, everolimus- and rapamycin-treated mice showed a reduction in the number of metastatic lymph nodes, resulting in an improvement of the overall survival of mice [44]. Molinolo et al. also reported the inhibitory effects of everolimus and rapamycin on xenografts derived from head and neck cancer cell line UDSCC2 in nude mice [31].

## 4. Clinical Trials on mTOR Inhibitors in Head and Neck Cancer

Although some studies have demonstrated the inhibitory effects of mTOR inhibitors on head and neck cancer using *in vitro* cell line model and *in vivo* xenograft model, little evidence has been obtained from clinical trials. Because most clinical trials were initiated from 2009 to 2011 and are recruiting participants, it is not strange that clinical trials have given little evidence. A phase I clinical trial showed that temsirolimus treatment resulted in partial response in a patient with head and neck cancer at stage T4N3M1 [45]. To evaluate the therapeutic effects of mTOR inhibitors on patients with head and neck cancer, a large number of clinical trials have been initiated (http://www.clinicaltrials.gov/) (Table 2).

## 5. Therapeutic Strategies of mTOR Inhibitors in Head and Neck Cancer

Since mTOR inhibitor alone has displayed inhibitory effects on head and neck cancer, it has potential as a single therapeutic agent. Furthermore, some studies have demonstrated that combined mTOR inhibitors with radiation, chemotherapeutic agents, or other targeted therapeutic agents resulted in synergistic repression on head and neck cancer. 

### 5.1. Evidence on Temsirolimus

Combined temsirolimus with radiotherapy displayed augmented inhibitory effects on tumor growth than radiation alone in mice bearing xenografts derived from head and neck cancer cell lines FaDu and SCC40. Moreover, combined temsirolimus with radiotherapy increased survival of mice with FaDu- and SCC40-derived xenografts. These results indicated that temsirolimus could be used in combination with radiotherapy to treat head and neck cancer [46]. Temsirolimus in combination with adriamycin/cisplatin resulted in synergistic suppression of cell proliferation of human head and neck cancer cell line KB and its multidrug-resistant subclone KB/7D by inhibiting the phosphorylation of 4EBP1 and S6K1 *in vitro* [47]. Temsirolimus repressed tumor growth more effectively than erlotinib (an EGFR inhibitor) in nude mice bearing xenografts derived from Detroit 562 cells (intermediate susceptibility to erlotinib), indicating that temsirolimus has potential as a therapeutic agent for EGFR-resistant head and neck cancer [43]. Temsirolimus augmented the inhibitory effects of the cetuximab-bevacizumab-irradiation combination in nude mice bearing xenografts derived from head and neck cancer cell line CAL33 [48]. 

### 5.2. Evidence on Everolimus

Everolimus displayed inhibitory effects on both cisplatin-resistant and cisplatin-sensitive NPC cell lines *in vitro* [49]. Combination of everolimus and cisplatin also exhibited synergistic repression on NPC cell lines [49].

## 6. Conclusion

In head and neck cancer, it has been shown that mTOR signaling pathway was activated, making it attractive for targeted therapy. Although some studies have demonstrated the inhibitory effects of mTOR inhibitors on head and neck cancer using *in vitro* cell line model and *in vivo* xenograft model, little evidence has been obtained from clinical trials. A large number of clinical trials have been initiated to evaluate the clinical application of mTOR inhibitors in patients with head and neck cancer. Combined mTOR inhibitors with radiation, chemotherapeutic agents, or other targeted therapeutic agents would result in synergistic repression on head and neck cancer, thus minimizing their toxicity and overcoming chemoresistant tumors. In addition, mTOR and downstream targets eIF4E, 4EBP1, S6K1, and S6 are potential diagnostic and prognostic biomarkers for head and neck cancer.

## Figures and Tables

**Table 1:** Chemotherapeutic model for head and neck cancer.

Chemotherapeutic agents	Treatment regimens	References
Platinum-based agents (cisplatin/carboplatin)	Single agent or in combination with 5-fluorouracil or taxane agents	[5]
Taxane agents (docetaxel/paclitaxel)	In combination with cisplatin or 5-fluorouracil	[6]
5-Fluorouracil	In combination with cisplatin or taxane agents	[7]

**Table 2:** Clinical trials of mTOR inhibitors in head and neck cancer.

Drugs	Treatment regimens	Study phase and disease status	Identifier no.
Rapamycin	Rapamycin once daily for 21 days followed by surgery	Phases I/II study in advanced head and neck cancer	NCT01195922
Temsirolimus	Temsirolimus with or without cetuximab	Phase II study in recurrent and/or metastatic head and neck cancer	NCT01256385
Temsirolimus	Temsirolimus	Phase II study in relapsed/recurrent head and neck cancer	NCT01172769
Temsirolimus	Temsirolimus, paclitaxel, and carboplatin	Phases I/II study in recurrent or metastatic head and neck cancer	NCT01016769
Temsirolimus	Temsirolimus, cisplatin, and cetuximab	Phases I/II study in recurrent or metastatic head and neck cancer	NCT01015664
Everolimus	Everolimus, docetaxel, and cisplatin	Phase I study in local-regional advanced head and neck cancer	NCT00935961
Everolimus	Carboplatin, cetuximab, and everolimus	Phases I/IIB study in recurrent metastatic head and neck cancer	NCT01283334
Everolimus	Everolimus, carboplatin, and paclitaxel	Phases I/II study in locally advanced head and neck cancer	NCT01333085
Everolimus	Everolimus, cetuximab	Phase I study in recurrent or metastatic head and neck cancer	NCT01637194
Everolimus	Everolimus	Phase II study in refractory, recurrent, and locally advanced head and neck cancer	NCT01051791
Everolimus	Everolimus, erlotinib	Phase II study in recurrent head and neck cancer	NCT00942734
Everolimus	Everolimus, placebo	Phase II study in locally advanced head and neck cancer	NCT01133678
